# Genome-Wide Association Study on the Development of Cross-Reactive Neutralizing Antibodies in HIV-1 Infected Individuals

**DOI:** 10.1371/journal.pone.0054684

**Published:** 2013-01-23

**Authors:** Zelda Euler, Marit J. van Gils, Brigitte D. Boeser-Nunnink, Hanneke Schuitemaker, Daniëlle van Manen

**Affiliations:** Department Experimental Immunology, Sanquin Research, Landsteiner Laboratory, and Center for Infectious Diseases and Immunity Amsterdam (*CINIMA*) at the Academic Medical Center of the University of Amsterdam, Amsterdam, The Netherlands; University of Alabama, United States of America

## Abstract

Broadly neutralizing antibodies may protect against HIV-1 acquisition. In natural infection, only 10–30% of patients have cross-reactive neutralizing humoral immunity which may relate to viral and or host factors. To explore the role of host genetic markers in the formation of cross-reactive neutralizing activity (CrNA) in HIV-1 infected individuals, we performed a genome-wide association study (GWAS), in participants of the Amsterdam Cohort Studies with known CrNA in their sera. Single-nucleotide polymorphisms (SNPs) with the strongest P-values are located in the major histocompatibility complex (MHC) region, close to MICA (P = 7.68×10^−7^), HLA-B (P = 6.96×10^−6^) and in the coding region of HCP5 (P = 1.34×10^−5^). However, none of the signals reached genome-wide significance. Our findings underline the potential involvement of genes close or within the MHC region with the development of CrNA.

## Introduction

An HIV-1 based immunogen that can elicit protective antibodies is not yet available. Considering the large variability of HIV-1, an ideal vaccine should be capable of eliciting antibodies that may be able to neutralize HIV-1 variants from different subtypes. This so-called cross-reactive neutralizing activity (CrNA) is observed in the natural course of infection in 10–30% of HIV-1 infected individuals [1]–[3]. While HIV-1 infected individuals have no clinical benefit from CrNA in their blood [2], broadly neutralizing antibodies can protect against infection when administered prior to inoculation [4]–[6]. Therefore, an immunogen that can elicit cross-reactive broadly neutralizing antibodies prior to infection is highly needed.

Until now, the clinical markers that best predict the development of CrNA are a high viral load and a reduced CD4^+^ T cell count, especially during primary infection [1], [2], [7], [8]. The capacity of the immune system to mount a protective response to pathogens after vaccination depends on several factors [9] and relies on complex cellular interactions that result in multiple outcomes. The reductionist approach to study separate components of this intricate system, although extremely valuable, can only give a narrow representation of the immune system. A systems genome-wide association (GWA) approach in natural infection can give a holistic view to understand the biological networks that drive the humoral response to make broadly cross-reactive neutralizing antibodies. Some studies have focused on host genetic factors and polymorphisms that associated with neutralizing antibody responses against other viruses or vaccinations. For example, the HLA class II DRB1*0701 allele was associated with failure to mount a neutralizing antibody response after influenza vaccination [10]. However, it remains unknown how human genetic variation might influence cross-neutralizing antibody responses after HIV-1 infection. This can ultimately provide valuable information for the design of an effective HIV-1 vaccine.

Here we investigated the role of the host genetic background on the ability to develop HIV-specific CrNA. By performing a GWAstudy, we identified associations between host genetic loci, especially in the major histocompatibility complex (MHC) region, and the presence and potency of HIV-1 specific CrNA in sera of participants of the Amsterdam Cohort Studies (ACS) on HIV-1 infection and AIDS.

## Methods

### Study Population

We studied HIV-1-infected homosexual men who participate in the Amsterdam Cohort Studies on HIV infection and AIDS (ACS), were enrolled in the cohort between October 1984 and March 1986, and from whom long-term follow-up data is available (every 3 months: collection of clinical and epidemiological data and cryopreservation of serum and peripheral blood mononuclear cells). In the first serum sample taken at entry in the cohort, 728 men tested negative for HIV-1 antibodies and 238 men tested positive for HIV antibodies of whom four refused to participate further; 131 of the initially negatively tested men subsequently seroconverted during active follow-up (until May 1996). For seroprevalent individuals, an imputed seroconversion date (on average, 18 months before entry into the ACS) was used [11]. AIDS-free survival was similar for persons who seroconverted during the cohort study and persons seroprevalent at entry (Log Rank *P* value >0.2), suggesting a good estimation of the seroconversion date in the latter group. The mean age at (imputed) seroconversion, as well as viral load and CD4^+^ T-cell count at set-point, was not different between both groups. Finally, heterozygosity for a 32 base-pair deletion in the *CCR5* gene had a similar effect on AIDS-free survival in the two cohorts [12]. Therefore, we here used the two cohorts as one study sample (*n* = 365). The mean age of participants at the time of (imputed) seroconversion was 34.5 years (range 19.5–57.7 years). From 335 of these 365 cohort participants a DNA sample was available for genotyping analysis and consequently these 335 individuals (205 seroprevalent cases and 130 seroconverters) were included in further analyses.

Sera were obtained on average at 34 months (range, 21–37 months) post imputed or documented date of seroconversion. Individuals from whom no serum sample was available between 21–45 months post seroconversion (n = 13), who received antiretroviral therapy (n = 2) or who did not pass the population stratification test (n = 28), as described below were excluded, leaving 292 individuals for analysis. The ACS are being conducted in accordance with the ethical principles set out in the declaration of Helsinki and all participants provided written informed consent. The study was approved by the institutional Medical Ethics Committee of the Academic Medical Center, University of Amsterdam.

### U87/Pseudovirus Assay for Testing of HIV-1 Cross-reactive Neutralizing Activity in Serum

Data on HIV-1 neutralizing activity in serum were partially available from our previous study (n = 110) [2], [7] and newly generated for 182 individuals. In short; sera were tested for cross-reactive neutralizing activity in a pseudovirus assay involving six tier two viruses in a single round of viral infection as developed by Monogram Biosciences. This six viral panel covered 93% of the variation in neutralization of a larger pseudovirus panel (n = 15) [3]. Previously we have shown that classification of CrNA in patient serum samples as determined on an independent 23 viral panel or on the smaller six viral panel used (included in the 23 viral panel) in this study, was highly correlated (Spearman r = 0.91, P<0.0001) [2]. The geometric IC_50_ value was calculated across the 6 viral panel per patient and ranked according to neutralization titer. Also the ranked number assigned according to the geometric IC_50_ value was highly correlated between those individuals tested on both the 23 viral panel or the smaller six viral panel (Spearmen r = 0.95, P<0.0001).

### Additional Phenotypes

AIDS according to the Centers for Disease Control (CDC) 1993 definition [13] was used as an endpoint in survival analysis. The other endpoint used in survival analysis was AIDS-related death, defined as death with AIDS-related malignancy, death with AIDS-opportunistic infections, or death with AIDS-related cause not specified by the treating physician. Viral load and CD4^+^ T-cell count at set-point was defined as the relatively steady level of HIV-1 RNA and CD4^+^ T-cell count at 18–24 months after seroconversion [14].

### HLA-typing

Genotyping of HLA loci was performed by sequence specific primers PCR.

### GWAS Scan Genotyping and Quality Control

DNA samples were genotyped using either Illumina’s Infinium HumanHap300 or Human 370CNV BeadChips (Illumina Inc, San Diego, USA). PLINK v1.07 [15] was used for quality control; Single-nucleotide polymorphisms (SNPs) (n = 21057) were excluded based on absence on one of the two BeadChips, call rate (<99%), minor allele frequency (<0.01) and Hardy-Weinberg Equilibrium deviation (<1E-6). Population stratification was addressed by PLINK (v1.07) by conducting identity-by-state (IBS) analysis. The first two dimensions of a multidimensional scaling analysis of IBS distances for each sample were calculated. We plotted the first two dimensions for each sample and removed non-Caucasian samples.

### Statistical Analysis

Association analysis between SNP genotypes and ranking order of individuals based on their geomean IC_50_ values of HIV-1-specific neutralizing activity was tested in a linear model assuming an additive model in PLINK software. Cox regression survival analysis was used to test association of SNPs with the course of HIV-1 infection, using AIDS and AIDS-related death as an endpoint. The association of the SNP with viral load set-point was tested using one-way analysis of variance (ANOVA).

### Heatmap Analysis Based on Cross-reactive Neutralizing Activity

A heatmap was made with a web-tool on the HIV database website (http://www.hiv.lanl.gov/content/sequence/HEATMAP/heatmap.html) which uses the heatmap tool “heatmap.2” of the gplots package of the statistical environment R (A Language and Environment for Statistical Computing), Kmeans clustering was performed on the rows and the columns and the rows/columns that fall in the same cluster are represented by the same colors on the row/column side bar. Bootstrapping was performed with 1000 iterations. Complete cluster analysis was performed on base10 data and output was given as a heatmap with a 9 colors “brewer” palette. Neutralizing IC_50_ titers were used to generate the heatmap.

## Results

### Study Population and Phenotype

**Figure 1:**
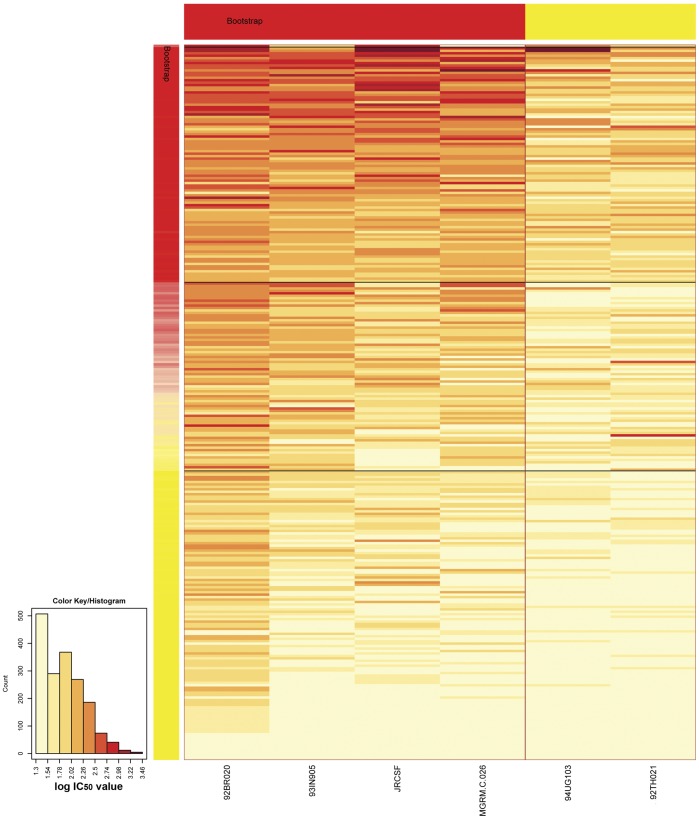
Heatmap and clustering analysis of HIV-1 specific cross-reactive neutralizing activity in serum. IC_50_ values of sera from 292 HIV-1 infected individuals at ∼35 months post (imputed) seroconversion (rows) against 6 viral isolates (columns) are shown. Darker colors represent more potent neutralization. Kmeans clustering was performed on the rows and the columns and the rows/columns that fall in the same cluster are represented by the same colors on the row/column side bar. Patients with cross-reactive neutralizing activity (CrNA) cluster together in the upper heatmap and patients with no CrNA cluster in the bottom of the heatmap.

To analyze the association between SNPs and the presence of CrNA in HIV-1 infected individuals, we first screened serum samples from participants of the ACS on HIV-1 infection and AIDS for the presence of CrNA. Participants were enrolled in the study when a serum sample was available ∼35 months (range 21–37 months) after seroconversion. The study population consisted of a total of 292 HIV-1 infected MSM from the ACS. Sera were screened for neutralizing activity on a panel of 6 viruses (**Figure 1**) and the patients were ranked on the geometric mean IC_50_ titer across this panel [median IC_50_ = 68 (range 20–782)] [3]. Geometric mean IC_50_ titers across the panel of six viruses were strongly correlated with both the number of viruses neutralized (Spearman r = 0.82, P<0.001) as well as the number of viruses that was neutralized with serum dilutions higher than 1∶100 (Spearman r = 0.91, P<0.001). Of the 292 individuals, 80 (27.4%) were able to neutralize the majority of viruses (≥4 viruses) at serum dilutions higher than 1∶100, 78 (26.7%) 2 or 3 viruses and 134 (45.9%) individuals’ sera neutralized 1 or no viruses. For optimal power in this study, all individuals were included in the GWAS and ranked based on geometric mean IC_50_ titers across the panel of six viruses.

### Genome-wide Association Analysis

**Figure 2:**
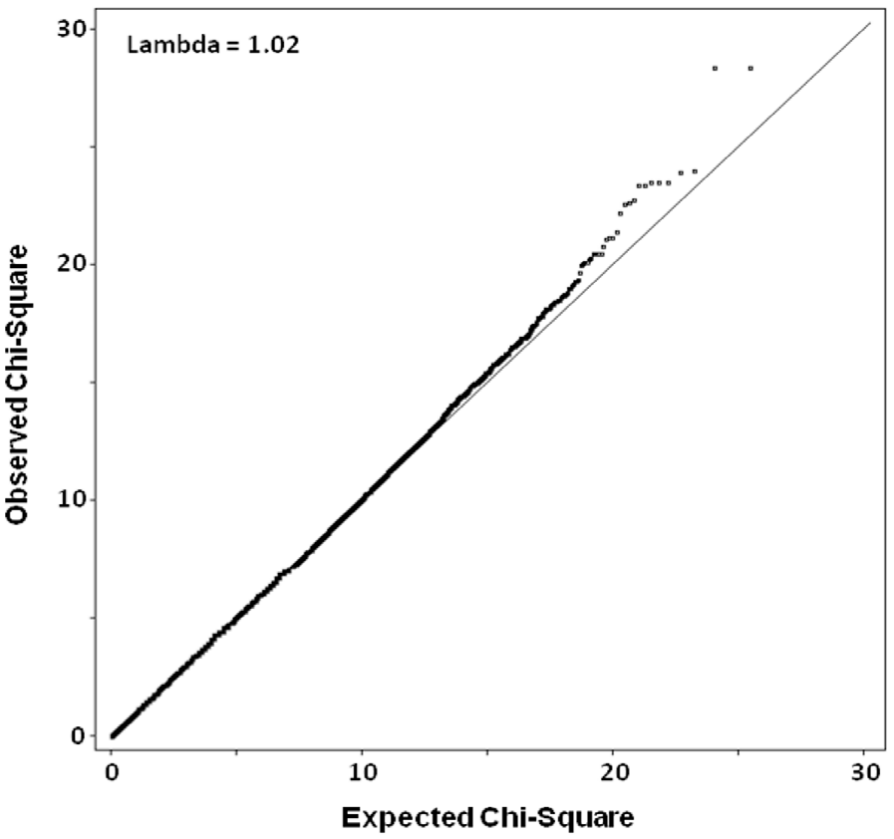
Quantile-Quantile Plot (Q-Q Plot) of P-values distribution from the GWAS on HIV-1 specific cross-reactive neutralizing activity. The comparison of the distribution of observed Chi-Square values against the theoretical model distribution of expected Chi-Square values is shown.

**Figure 3:**
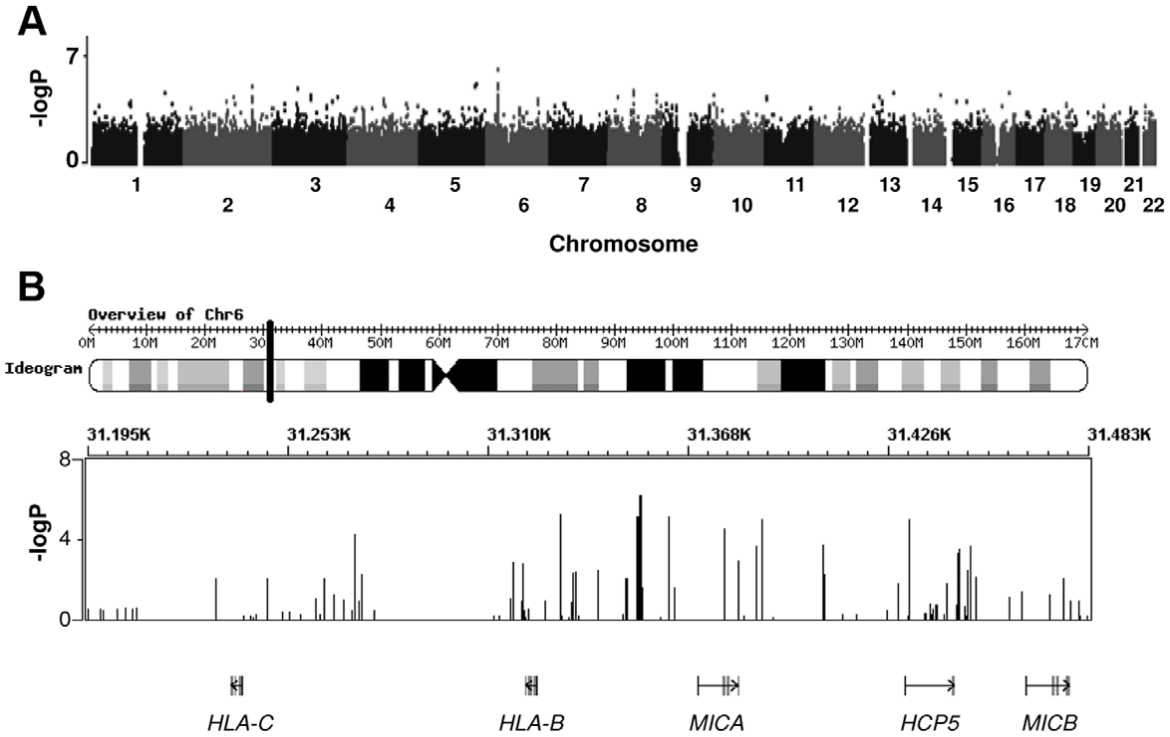
Genome overview of P-values for associations of SNPs with HIV-1-specific cross-reactive neutralizing activity. Genome overview (A) and Major Histocompatibility Complex region (B).

Associations between 296,446 SNPs and the rank of 292 individuals, assigned to them on the basis of their geometric mean IC_50_ titers in serum across the panel of six viruses, were tested using a linear regression model assuming an additive model. The Quantile-Quantile Plot (Q-Q plot, **Figure 2**) revealed that there was no inflation of false positive results (λ = 1.02). **Figure 3a** shows the distribution of the P-values along the genome and in **Table 1** the top signals with a P-value below 2.0×10^−5^ are shown. Albeit that none of the signals were genome-wide significant after correction for multiple testing, some interesting signals were identified.

**Table 1:** P-values for top ranking associations of SNPs with HIV-1-specific cross-reactive neutralizing activity, as well as P-values after correction for viral load and CD4^+^ T-cell count at set-point as covariates.

Univariate analysis							Covariates	
SNP	P-value	Chr	Coordinate	Gene	Position	Alleles	Viral load setpoint	CD4 setpoint
rs13437082	7.68E−07	6	31354560	MICA	16 kb 5′	A/G	9.37E−06	6.22E−04
rs4711269	7.68E−07	6	31354819	MICA	16 kb 5′	A/G	9.37E−06	6.22E−04
rs10045068	6.72E−06	5	155081309	intergenic		G/A	6.54E−06	3.25E−04
rs2523554	6.96E−06	6	31331829	HLA-B	7 kb 5′	G/A	2.37E−05	9.84E−05
rs2428486	8.68E−06	6	31353593	MICA	16 kb 5′	G/A	6.64E−05	3.52E−03
rs2523467	8.68E−06	6	31354104	MICA	17 kb 5′	A/G	6.64E−05	3.52E−03
rs2844529	8.68E−06	6	31362930	MICA	28 kb 5′	A/G	6.64E−05	3.52E−03
rs2165172	9.08E−06	2	184800983	intergenic		A/G	9.59E−05	1.48E−05
rs10079250	9.23E−06	5	149450132	CSF1R	Coding	G/A		
rs2844511	1.27E−05	6	31389784	MICA	7 kb 3′	A/G	4.04E−05	4.88E−05
rs2284178	1.34E−05	6	31432125	HCP5	Coding	A/G	8.27E−06	4.44E−04

Chr; chromosome.

This GWAS pointed specifically to the major histocompatibility complex (MHC) gene region on chromosome 6 to be potentially associated with the potency of CrNA in serum. Indeed, there is a clear overrepresentation of SNPs in the MHC class I chain-related protein A (MICA), HLA-B, and HLA complex P5 (HCP5) gene region among the top associations with CrNA (**Figure 3b**). The two top SNPs rs13437082 and rs4711269, located 16 kb upstream of MICA, are in perfect LD, with a P = 7.68×10^−7^ (**Table 1**) for the association with the potency of CrNA. Three other SNPs, (rs2428486, rs2523467 and rs2844529), were also closely located to MICA and in perfect LD with each other. These three SNPs were in moderate LD (r^2^ = 0.7) to the two top SNPs in this GWAs. SNP rs2844511, located 7 kb upstream of MICA, has a P-value of 1.27×10^−5^ for the association with the potency of CrNA. This SNP seems to be an independent marker for the association of the MICA region with CrNA, since rs284511 is not in high LD (r^2^<0.4) with the five SNPs located downstream of MICA.

SNP rs2284178 causes an amino acid change in the HCP5 gene region and this coding SNP seems to be associated with the presence of CrNA against HIV-1 (P = 1.34×10^−5^, **Table 1**). This SNP is not in high LD (r^2^<0.3) with SNP rs2395029 in the HCP5 gene region, which was previously identified to be strongly associated with HIV-1 viral load [16] and disease non- progression [17]. Finally, the minor allele of SNP rs10079250, which is located in the coding region of colony stimulating factor 1 receptor (CSF-1R) on chromosome 5, has a P-value of 9.23×10^−6^ for the association with CrNA.

Viral load and CD4 counts have shown to be strongly associated with the presence of CrNA [1], [2], [7], [8]. Also in this cohort viral load at setpoint (Spearman r = 0.18, P = 2.0×10^−3^, data not shown) and CD4 count at setpoint (Spearman r = −0.24, P<0.0001, data not shown) were associated with geomean IC_50_ titers. In order to assess whether viral load and CD4 count at setpoint influenced associations with the top ranked SNPs, a covariate analysis was performed with both these factors as covariates in our GWAS on CrNA (**Table 1**). SNP rs2284178 is located in the coding region of HCP5, and was found to be associated with CrNA in this present study. This SNP is not in high LD (r^2^<0.3) with the minor allele of SNP rs2395029 in HCP5, which was originally identified to be associated with controlled HIV-1 load via linkage with HLA-B57 [16]. Interestingly, the association of SNP rs2284178 with CrNA was even stronger after correction for viral load (P = 8.27×10^−6^, **Table 1**). Also, the association of SNP rs13437082 with CrNA is only partially dependent on viral load after correction for viral load (P = 9,37×10^−6^, **Table 1**).

Previously we have shown that the presence of CrNA in serum did not associate with the clinical course of infection [2]. Some of the genes that are linked to the SNPs with a top ranking association with CrNA in serum, were previously described to be associated with HIV-1 viral load and disease progression, for example the minor allele of HCP5 SNP rs2395029 that was associated with a lower viral load and delayed disease progression after infection [14], [16], [18]–[21]. We wanted to exclude that these SNPs were indeed not surrogate markers for disease progression. However, none of the SNPs that associated with CrNA were associated with disease progression in this cohort (**Table 2**).

**Table 2:** P-values for different endpoints in survival analysis and viral load at set-point of top ranking SNPs associated in GWA study with HIV-1-specific cross-reactive neutralizing activity.

			Survival analysis		
SNP	Gene	Position	AIDS	Death	Viral load setpoint
rs13437082	MICA	16 kb 5′	6.00E−01	4.00E−01	5.50E−01
rs4711269	MICA	16 kb 5′	6.00E−01	4.00E−01	5.50E−01
rs10045068	intergenic		5.40E−01	5.40E−01	6.20E−01
rs2523554	HLA−B	7 kb 5′	6.00E−02	3.50E−01	6.00E−02
rs2428486	MICA	16 kb 5′	3.80E−01	3.90E−01	9.50E−01
rs2523467	MICA	17 kb 5′	3.80E−01	3.90E−01	9.50E−01
rs2844529	MICA	28 kb 5′	3.80E−01	3.90E−01	9.50E−01
rs2165172	intergenic		3.20E−01	2.60E−01	3.00E−02
rs10079250	CSF1R	Coding	3.90E−01	8.90E−01	6.80E−01
rs2844511	MICA	7 kb 3′	3.00E−01	5.40E−01	4.30E−01
rs2284178	HCP5	Coding	4.50E−01	7.00E−01	6.70E−01

### Associations between HLA and CrNA

The overrepresentation of signals in the MHC region on chromosome 6 in the GWAS on HIV-1-specific neutralizing activity prompted us to test for a potential association between the different HLA alleles present in the study population and CrNA. After correction for the number of HLA-alleles tested, only the prevalence of HLA-B*57 was found to be significantly decreased in individuals with broadly neutralizing activity in the ACS (P = 1.5×10^−7^). The prevalence of HLA-B*07 was enriched in individuals with CrNA, albeit that this association was no longer significant after correction for multiple-testing (data not shown). No significant associations between HLA-C alleles, or HLA class II alleles and CrNA were observed (data not shown).

## Discussion

The role of host genetic markers in HIV-1-specific CrNA remains largely unknown. In our present study we performed a GWAS in HIV-1 infected individuals of the ACS on the neutralization titers of CrNA in serum at ∼35 months post seroconversion. The prevalence of CrNA in this cohort (27%) was similar to previously described cohorts [1], [3], [22]–[26].

Although none of the signals reached genome-wide significance, our GWAS revealed a considerable number of SNPs within the MHC region to be potentially associated with CrNA in HIV-1 infected individuals. Six of the SNPs that ranked in the top of associations with CrNA are located in the MICA gene region on chromosome 6, in two separate LD blocks. MICA is distant, but a structural homolog of MHC class I molecules, and a ligand for NKG2D, which is expressed on natural killer (NK) cells, most NKT cells, all CD8 T cells and subsets of γδ T cells and CD4 T cells [27]. NKG2D is a potent activating NK cell receptor and it has been shown that the antiviral factor APOBEC3G and HIV-1 Vpr both upregulate NKG2D-ligands on HIV-1 infected cells, enhancing NK-cell mediated killing [28], [29]. Furthermore, elevated levels of soluble MICA have been shown to impair NK cell function in chronic HIV-1 infection [30]. MICA is also a ligand for NKG2D on γδ T cells, which have been shown to increase neutralizing antibodies in the absence of CD4 αβ T cells against vesicular stomatitis virus (VSV), foot-and-mouth disease virus, as well as *Borrelia burgdorferi,* the causative agent for lyme disease [31]–[33].

SNP rs2284178 which was found to be associated with CrNA in this study is located in the coding region of HCP5. However, it is not in high LD with the minor allele of SNP rs2395029 identified to be associated with HIV-1 viral load control via linkage with HLA-B57 [16]. Furthermore, the association of SNP rs2284178 with CrNA was even stronger after correction for viral load, suggesting that this polymorphism does not exert its role in the emergence of CrNA solely through controlling viral load, even though the geometric mean IC_50_ titer of sera across the viral panel did associate with viral load at set-point.

The outcome of our GWAS prompted us to compare the prevalence of all HLA-types with the CrNA neutralization titers in these individuals. Conversely to the association between HLA-B*57 and lower CrNA titers, the HLA-B*07 allele was more prevalent among individuals with higher titers of CrNA, although not significant after correction for multiple testing. Interestingly, HLA-B*07, which is part of the HLA-B7 supertype, is more prevalent among HIV-1 infected individuals who experience a more rapid disease progression and a higher viral load at set-point [34]. The higher prevalence of a non-protective HLA-B type and the lower prevalence of protective HLA-B types in individuals with higher CrNA titers fit the observations that CrNA does not protect from HIV-1 disease progression [2], [8]. This observation is further strengthened by the fact that none of the top SNPs for CrNA was associated with disease progression in the ACS.

SNP rs10079250 in the coding region of CSF-1R, seems to be associated with CrNA. This gene was identified in a recent genome-wide analysis to play a role in HLA class II restricted antigen presentation [35]. In our study no HLA class II alleles were significantly associated with CrNA. However, other genes involved in the HLA class II antigen presentation process may still be involved. CSF-1R, the natural ligand of this receptor, controls the production, differentiation and function of macrophages which may indirectly influence B-cell functioning. However, the exact mechanism by which this SNP in CSF1R may influence CrNA remains to be established.

The results from our present study are based on HIV-1 infected MSM from the Netherlands, infected with HIV-1 subtype B. The identified signals should be replicated in other cohorts to confirm their association with HIV-1 specific CrNA. In addition, it may be interesting to see if these SNPs are associated with the quality of humoral immunity in general.

In conclusion, we have identified several host genetic factors that may potentially be associated with the development of HIV-specific CrNA in serum collected at approximately 35 months post-seroconversion from HIV-1 infected individuals. Although genome-wide significance was not reached, the highest associations were seen for SNPs in or near genes in the HLA-region that may influence the eliciting of CrNA, which might be partially antigen load dependent.

These results indicate that a comprehensive analysis at the system-level can greatly facilitate the screening for host factors that associate with neutralizing responses in natural infection, This may provide clues for the optimalization of antigens and antigen formulations (i.e. adjuvanted antigens) to induce the immune pathways that are required for the desired adaptive immune response.
